# Supplementary material for: DMRforPairs: identifying Differentially Methylated Regions between unique samples using array based methylation profiles
Source: BMC Bioinformatics. 2014 May 15;15:141. doi: 10.1186/1471-2105-15-141 (PMC4046028; doi:10.1186/1471-2105-15-141)
Supplement: Additional file 2 — DMRforPairs output for the comparison of A431-MCF7 and NA17018-NA17105. Please start from the HTML files in each folder. Available via the BMC Bioinformatics website. [file 1471-2105-15-141-S2.zip › 1394847754114233_MOESM2_ESM/A431_MCF7/figures/23777.pdf]

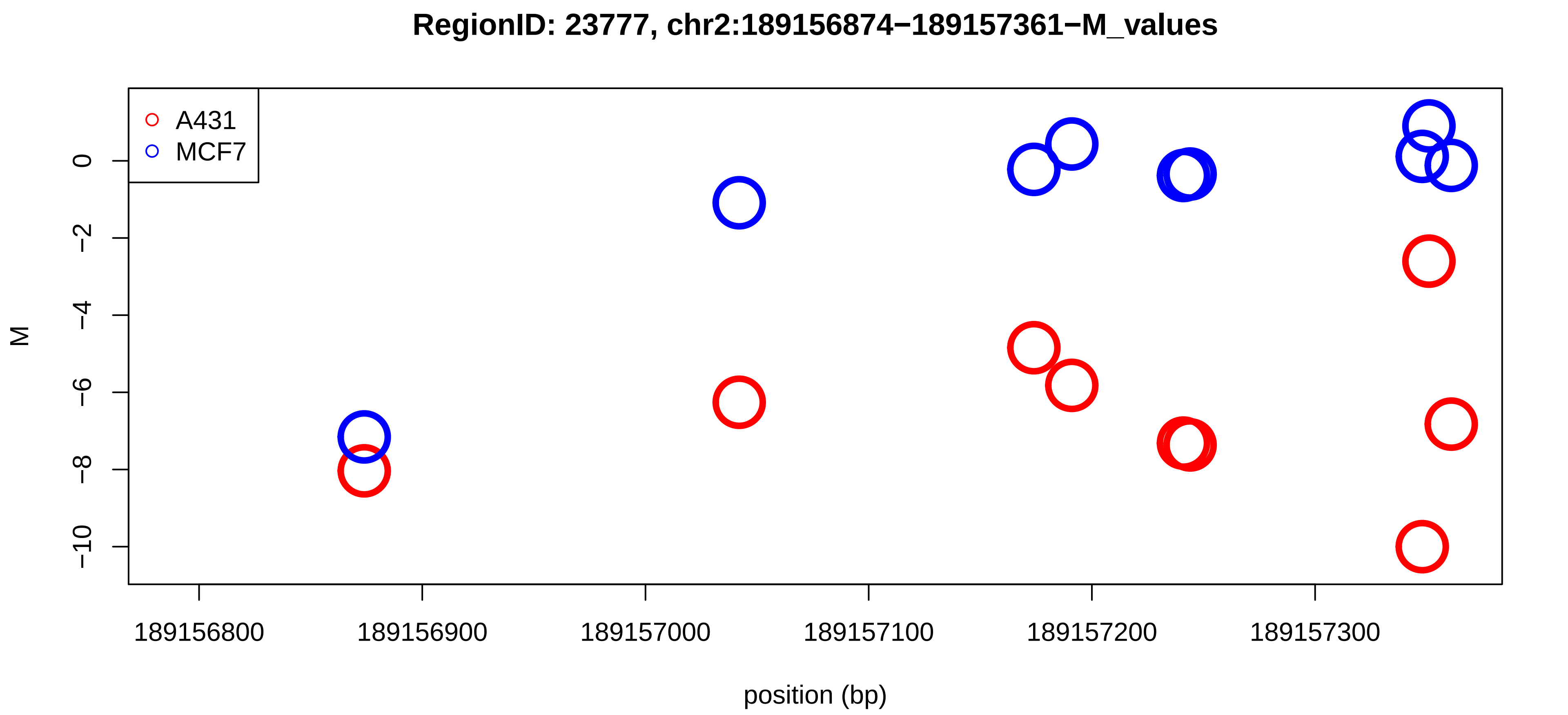
RegionID: 23777, chr2:189156874−189157361−M_values
A431
MCF7
M
0
−2
−4
−6
−8
−10
189156800
189156900
189157000
189157100
189157200
189157300
position (bp)

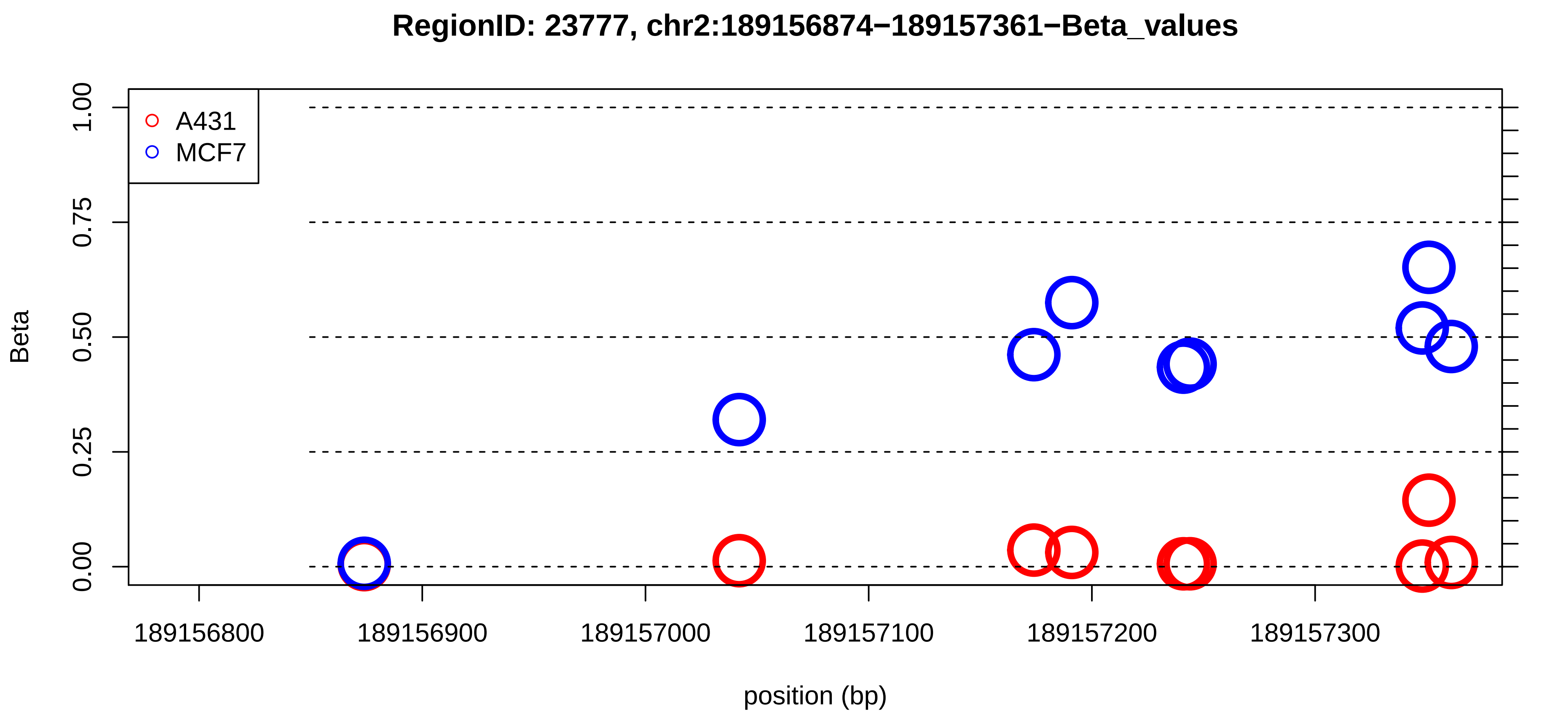
RegionID: 23777, chr2:189156874−189157361−Beta_values
A431
MCF7
Beta
1.00
0.75
0.50
0.25
0.00
189156800
189156900
189157000
189157100
189157200
189157300
position (bp)

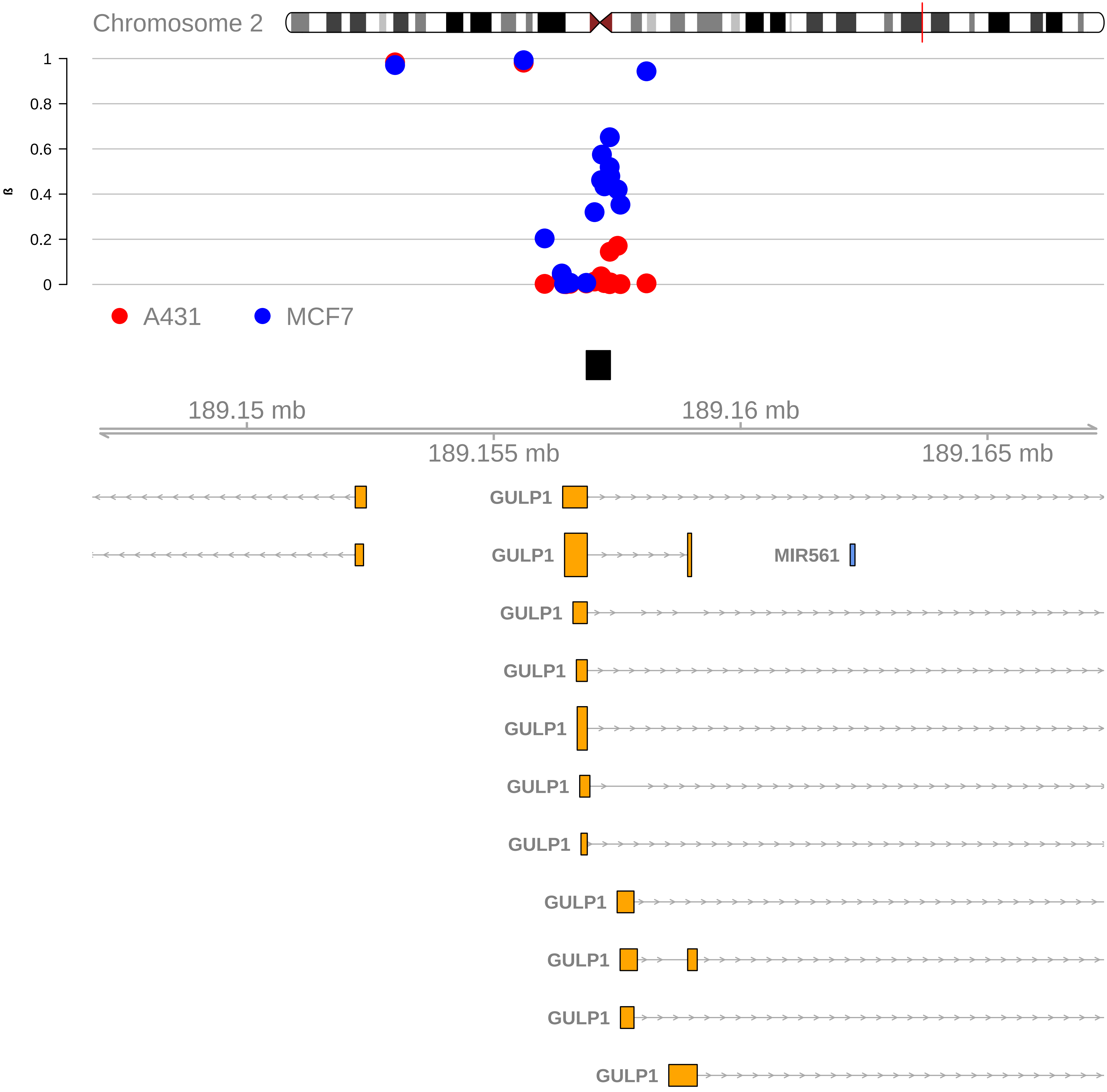
Chromosome 2
ß
1
0.8
0.6
0.4
0.2
0
A431
MCF7
189.15 mb
189.16 mb
189.155 mb
189.165 mb
GULP1
GULP1
MIR561
GULP1
GULP1
GULP1
GULP1
GULP1
GULP1
GULP1
GULP1
GULP1
